# Silicon nanocrystal hybrid photovoltaic devices for indoor light energy harvesting†

**DOI:** 10.1039/d0ra00804d

**Published:** 2020-03-27

**Authors:** Munechika Otsuka, Yuki Kurokawa, Yi Ding, Firman Bagja Juangsa, Shogo Shibata, Takehito Kato, Tomohiro Nozaki

**Affiliations:** School of Engineering, Tokyo Institute of Technology 2-12-1 O-okayama, Meguro-ku Tokyo 152-8550 Japan nozaki.t.ab@m.titech.ac.jp; Department of Mechanical Engineering, National Institute of Technology, Oyama College 771 Nakakuki, Oyama Tochigi 323-0806 Japan; Institute of Photoelectronic Thin Film Devices and Technology, Nankai University Tianjin 300071 PR China; Faculty of Mechanical and Aerospace Engineering, Institut Teknologi Bandung Ganesha Street No. 10 Bandung 40132 Indonesia

## Abstract

Silicon nanocrystals (SiNCs) featuring size-dependent novel optical and electrical properties have been widely employed for various functional devices. We have demonstrated SiNC-based hybrid photovoltaics (SiNC-HPVs) and proposed several approaches for performance promotion. Recently, owing to the superiorities such as low power operation, high portability, and designability, organic photovoltaics (OPVs) have been extensively studied for their potential indoor applications as power sources. SiNCs exhibit strong light absorption below 450 nm, which is capable of sufficient photocurrent generation under UV irradiation. Therefore, SiNC-HPVs are expected to be preferably used for energy harvesting systems in indoor applications because an indoor light source consists of a shorter wavelength component below 500 nm than solar light. We successfully demonstrated SiNC-HPVs with a PCE as high as 9.7%, corresponding to the output power density of 34.0 μW cm^−2^ under standard indoor light irradiation (1000 lx). In addition, we have found that SiNC defects working as electron traps influence the electrical properties of SiNCs substantially, a thermal annealing process was conducted towards the suppression of defects and the improvement of the SiNC-HPVs performance.

## Abstract 2

The potential of SiNC-based hybrid photovoltaics is opened up to be used for the IoT-related energy harvesting of indoor applications.

## Graphical abstract

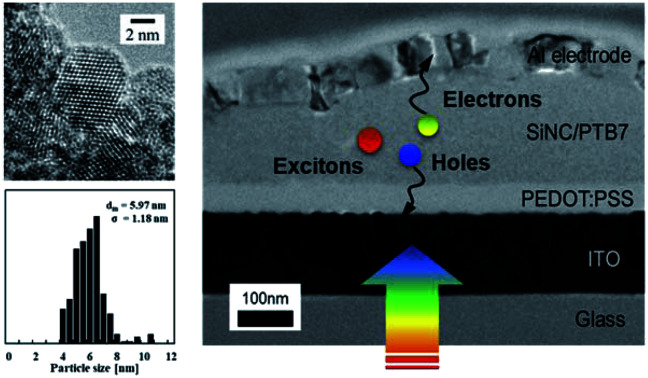

## 1. Introduction

Nanomaterials with sizes down to a single nano-order have been well-known to have size-dependent novel optical and electrical properties that appear different from those of their bulk structures.^1–6^ Among them, silicon nanocrystals (SiNCs) have attracted much attention as nontoxic and abundant semiconducting materials. The application of SiNCs has also been studied in numerous devices,^7–10^ and SiNCs allow expansion of the flexibility of device design and provide optimum performance. High-performance organic semiconducting materials with various light absorption capabilities have been developed, and interest in organic photovoltaics (OPVs) has increased over the past decade as an important energy harvesting technology.^11–16^ OPVs have been studied to meet various demands, such as color and shape design and transparency of devices by optimizing the molecular design of organic semiconducting materials, which is an advantage not found in other solar cells.

Previously, we reported a synthesis method for free-standing and narrow-size-distribution SiNCs by nonthermal plasma CVD^17–19^ and demonstrated that SiNC-based hybrid photovoltaics (hereafter, SiNC-HPVs) have a power conversion efficiency (PCE) of 3.6% under standard solar irradiation (AM 1.5 G 100 mW cm^−2^).^20–23^ SiNCs are expected to possess size-tunable photoabsorbance capability from near IR (bulk; 1.1 eV) to near UV (nanocrystals; 3–7 eV),^24–26^ enabling all-silicon tandem PVs with PCEs of more than 30%.^27^ Although photoluminescence (PL) exhibited clear tunability in terms of crystal size, SiNCs showed a negligible dependence on size tunable absorption in the visible light spectrum. Additionally, SiNCs exhibited strong light absorption below 450 nm, meaning that the level of the interband transition is not size dependent.^1,28^ Therefore, SiNC-HPVs are expected to be used for energy harvesting systems of indoor light sources because LEDs and fluorescent lamps consist of more wavelength components below 500 nm than solar light.^28–30^ In recent years, due to the rapid development of IoT (Internet of Things) related technology, the number of IoT devices has dramatically increased.^31,32^ Many type of printable PV devices such as OPVs,^33–42^ dye-sensitized solar cells^43,44^ and perovskite solar cells^45,46^ have been explored for indoor applications, OPVs are especially expected to be used as an energy harvesting system for IoT devices because of their potential for low power operation, independent and distributed applications, high portability and device designability. In this study, we successfully demonstrated SiNC-HPVs with PCE as high as 9.7% and power density of 34.0 μW cm^−2^ under standard indoor light irradiation (1000 lx). A tandem device is indeed an attractive approach for providing the voltage to drive off-grid electronic devices in practical application. Meantime, a serial connection of multiple devices is readily applicable with minimum cost investment which is also attractive from a practical viewpoint. At the moment we focus on the single device performance which is beneficial for the optimum design of tandem device architecture. First, we describe the thermal annealing effects for SiNC properties and their application for SiNC-HPVs under the simulated solar light illumination. Subsequently, performance measurements under standard indoor light are described. Finally, concluding remarks are provided.

## 2. Experimental section

### 2.1 SiNC synthesis

SiNCs were synthesized by nonthermal plasma CVD. A mean particle size of 6 nm with a narrow size distribution was applied for the device fabrication. Experimental conditions were determined and explained in detail in previous works.^17–19^ Briefly, SiCl_4_ is employed as a precursor material because it is abundant, nontoxic and inexpensive. A mixture of SiCl_4_, H_2_, and Ar flows through a quartz tube reactor where capacitively-coupled nonthermal plasma is generated using a very-high-frequency (70 MHz) power source. Plasma-activated H_2_ abstracts chlorine from SiCl_4_, leading to a nucleation and subsequent growth of SiNCs.

### 2.2 Annealing SiNCs

Before blending the SiNCs into a polymer solution, SiNCs were employed for thermal annealing to reduce crystal defects.^47,48^ Crystal defects of SiNCs are detrimental because defects trap photogenerated carriers and deteriorate carrier mobility. As-produced SiNCs with chlorine-terminated surfaces were exposed to ambient air for 1 hour, producing silicon suboxide surfaces to remove surface chlorine. Subsequently, SiNCs were subjected to hydrofluoric acid vapor (HF) dry etching for 48 hours using a 50% HF/water solution in a sealed container, producing fully hydrogen-terminated SiNCs. The SiNCs were heated to 200 °C and 400 °C for 1 hour in a flow-type tubular reactor using pure hydrogen at 100 kPa. The amount of defects was evaluated semiquantitatively by electron spin resonance (ESR, JES-FA100; JEOL Ltd.); 20 mg of hydrogen-terminated SiNCs was packed into plastic tubes for the ESR measurement. The surface structure of hydrogenated SiNCs (Si–H_*n*_: *n* = 1–3) was analysed by attenuation total reflection Fourier-transform infrared (ATR-FTIR) spectroscopy (FT/IR-6100; JASCO Corp.).

### 2.3 Device fabrication and performance analysis

**Fig. 1:**
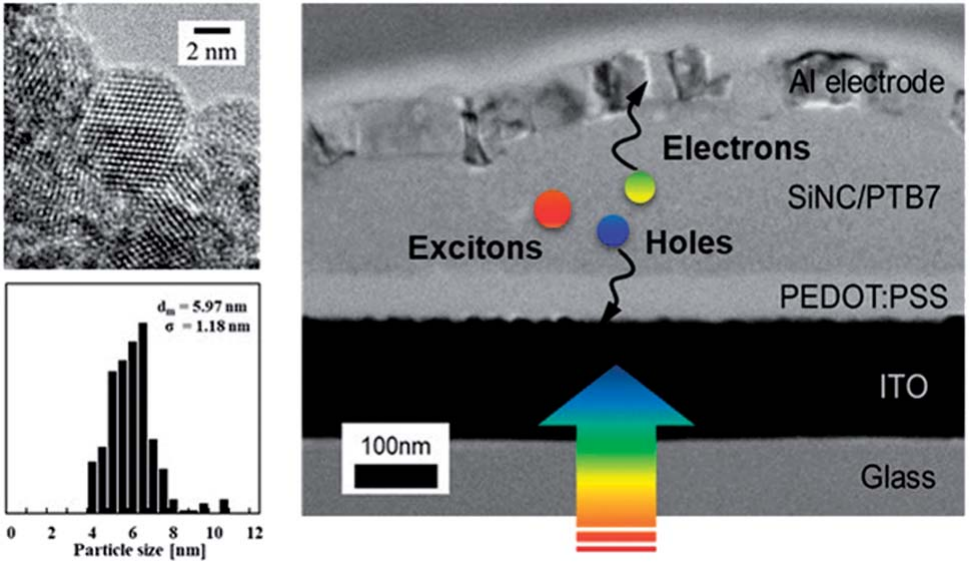
Cross-section TEM micrograph of SiNC-HPV device. The inset shows TEM micrograph of SiNCs and their size distribution.

**Fig. 2:**
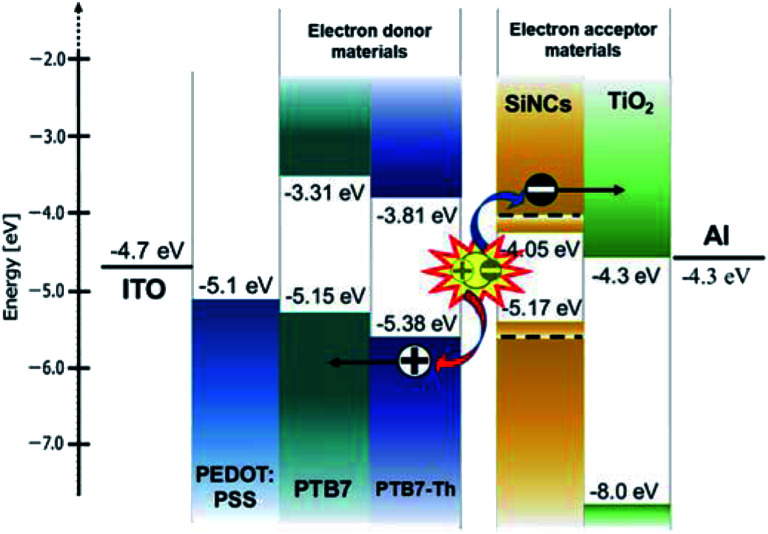
Energy diagram of hybrid photovoltaic devices.

SiNC-HPVs were fabricated by a layer-by-layer approach in a nitrogen-purged glove box (<1 ppm oxygen and water; Miwa Mfg Co., Ltd.). A bulk-heterojunction type photoactive layer is produced by SiNCs and p-type semiconducting polymer blended solution. Here, the polymer represents either poly({4,8-bis[(2-ethylhexyl)oxy]benzo[1,2-*b*:4,5-*b*′]dithiophene-2,6-diyl}{3-fluoro-2-[(2-ethylhexyl)carbonyl]thieno[3,4-*b*]thiophenediyl}) (PTB7, Sigma-Aldrich) or poly([2,6′-4,8-di(5-ethylhexylthienyl)benzo[1,2-*b*;3,3-*b*]dithiophene]{3-fluoro-2[(2-ethylhexyl)carbonyl]thieno[3,4-*b*]thiophenediyl}) (PTB7-Th, Sankyo Kagaku Yakuhin Co.). Visible photo-absorption occurs in the polymer matrix, and photogenerated electron–hole pairs are separated at SiNC and polymer interfaces. The SiNC network provides electron transport pathways, while the polymer matrix serves as the hole transport network. The cross-sectional transmission electron microscope image is shown in Fig. 1 (TEM, JEM-2010F; JEOL Ltd.). Fig. 2 shows the energy diagram of the device with the structure of glass/ITO/PEDOT:PSS/(SiNCs + polymer)/Al. The values of the valence and conduction band edges of the SiNCs are enlarged from the bulk values due to the quantum confinement effect as represented by dotted lines.^24–26^ Indium-tin oxide (ITO) coated glass with a resistance of 15 Ω □^−1^ was employed as the substrate, patterned with photolithography and a wet-etching process. The patterned ITO substrate was treated by ultrasonication in acetone and deionized water for 30 min before film fabrication by a spin-coating process. Poly(3,4-ethylenedioxylenethiophene):poly(styrenesulphonic acid) (PEDOT:PSS, CLEVIOS PH1000, Heraeus) was spin-coated as a hole transporting layer (HTL) at 4000 rpm for 1 min and annealed at 130 °C for 10 min. The SiNCs and semiconducting polymer (PTB7, PTB7-Th) were dissolved respectively in chlorobenzene with a predetermined ratio and homogenized for a stable solution. The SiNC solution was mixed with polymer ink at a volume ratio of 1 : 1 and stirred for 24 hours before use. The blended solution was spin-coated at 900 rpm for 90 seconds on the HTL to form a photoactive layer with a thickness of *ca.* 150 nm. Finally, an aluminium electrode with a thickness of 80 nm was evaporated on the top. One substrate has 6 independent PV cells with an active area of 4.6 mm^2^ to confirm the variation and reproducibility of the photovoltaic performance. The photocurrent density and voltage (*J*–*V*) characteristics were measured using a digital current–voltage source metre (Keithley 2400; Tektronix) under illumination with AM 1.5 G simulated solar light at 100 mW cm^−2^ (1 sun), where the illumination of 116 200 lx was measured (HAL-320; Asahi Spectra Co., Ltd.). Additionally, LED light source simulated standard indoor light (BLD-100; Bunkoukeiki Co., Ltd.) was used for the characterization of the photovoltaic performance.

## 3. Results and discussion

### 3.1 SiNC defect control by thermal annealing

**Fig. 3:**
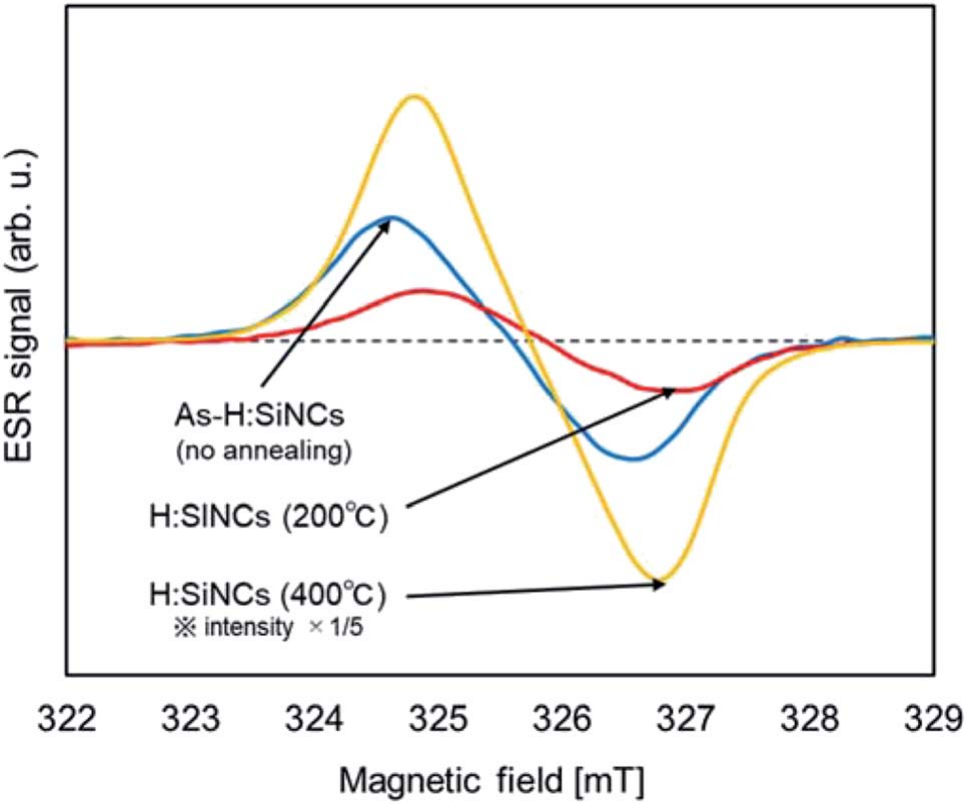
ESR spectra of SiNCs before and after thermal annealing; As-H:SiNCs (no annealing), H:SiNCs (200 °C for 1 hour) and H:SiNCs (200 °C for 1 hour).

**Fig. 4:**
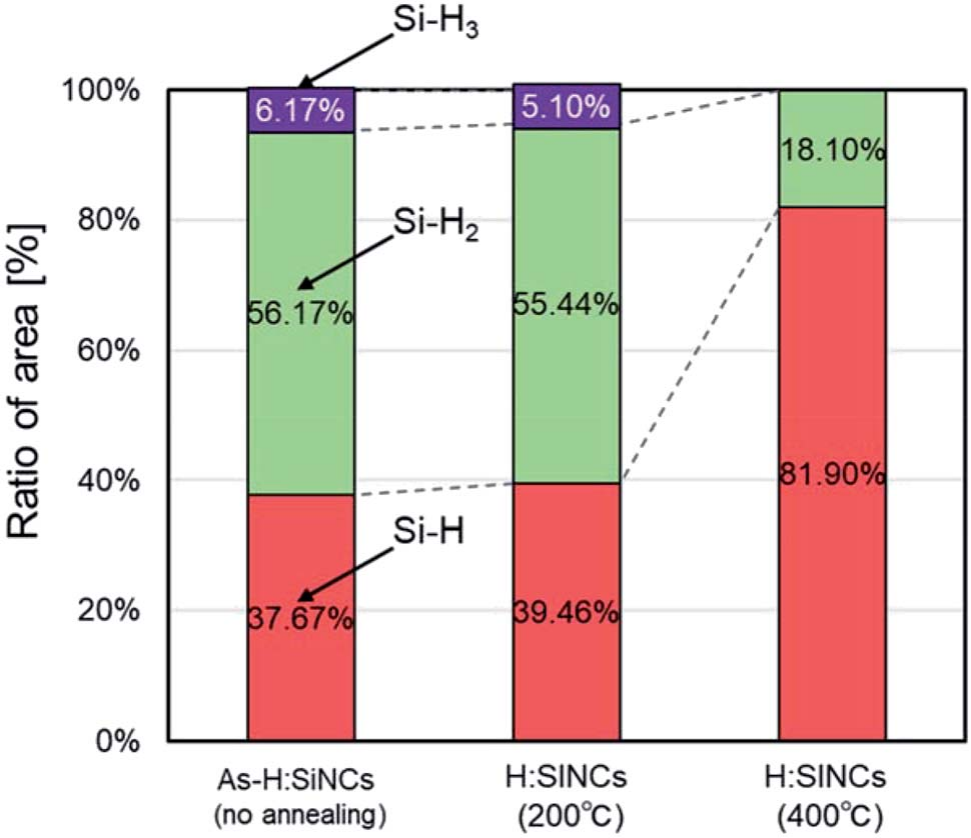
Ratio of hydrogen groups on SiNC surface.

Fig. 3 shows the ESR spectra of SiNCs annealed at temperatures of 200 °C and 400 °C for 1 hour. Semiquantitative values of the amount of defects were obtained by double integration of the resonance peak. The reduction in the amount of defects by nearly 70% of the as-etched SiNCs was confirmed at an annealing temperature of 200 °C. The change in the surface structure was not confirmed by ATR-FTIR analysis between as-etched and 200 °C annealing (Fig. S1†), indicating that low-temperature annealing (200 °C) can reduce the internal crystal defects; however, the reconstruction of the crystal structure does not fully eliminate disordered Si–Si bonding. A significant increase in defects is observed from the 400 °C annealing sample. This is caused by the desorption of surface hydrogen occurred at 400 °C annealing. Hydrogen-terminated SiNCs have Si–H, Si–H_2_, and Si–H_3_ bonds on the surface. Among them, the Si–H_3_ bond is unique to SiNCs. Three peaks show the stretching vibrations of Si–H (2087 cm^−1^), Si–H_2_ (2108 cm^−1^), and Si–H_3_ (2142 cm^−1^) bonds wavenumbers near 2000 cm^−1^.^49^ The deconvolution of Si-H_*n*_ absorption spectrum was performed assuming a Lorentzian profile, and the result is shown in Fig. S2.† The change in the ratio of hydrogen groups on the SiNCs surface by thermal annealing are summarized in Fig. 4. When SiNCs were annealed at 400 °C, hydrogen in Si–H_3_ was fully desorbed and the fraction of Si–H_2_ decreased. The activation energy of Si–H_*n*_ desorption have been reported to be 2.54 ± 0.04 eV (Si–H), 1.9 ± 0.1 eV (Si–H_2_), and 1.8 ± 0.1 eV (Si–H_3_), respectively.^50^ Hydrogen desorption occurs at bonds with lower activation energy which explains our observation shown in Fig. 4, S1 and S2.†

### 3.2 Photovoltaic performance

**Fig. 5:**
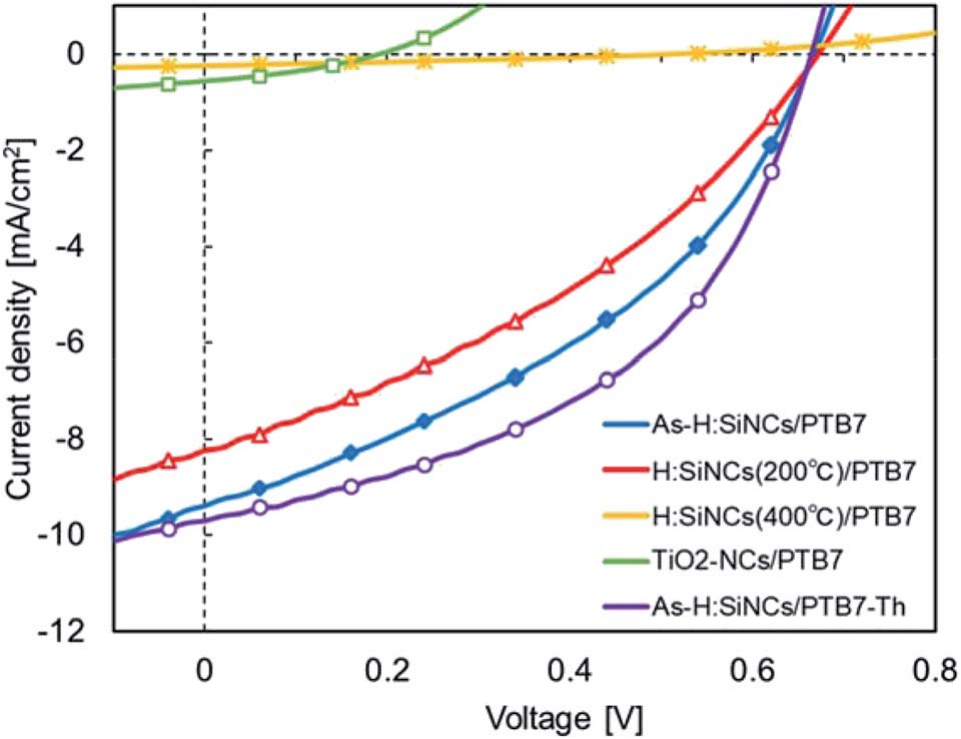
*J*–*V* characteristics of hybrid photovoltaics under standard solar irradiation (1 sun).

The *J*–*V* characteristics for devices fabricated using as-etched SiNCs, low-defect SiNCs (annealed at 200 °C), and high-defect SiNCs (annealed at 400 °C) under 1 sun illumination are shown in Fig. 5, and the corresponding performance parameters are summarized in Table 1. PTB7 was used as a p-type semiconducting polymer. An energy conversion efficiency (PCE) of 2.44% with a short-circuit current density (*J*_SC_) of 9.39 mA cm^−2^ was achieved on the device with as-etched SiNCs. Although low-defect SiNCs are expected to improve the electron mobility,^21,22,48^*J*_SC_ slightly decreased to 8.24 mA cm^−2^. The *J*_SC_ of the device with high-defect SiNCs dramatically dropped to 0.24 mA cm^−2^. Meanwhile, the fill factor (FF) for SiNCs/PTB7 devices is unexpectedly low. Dissociation of photo-generated excitons occurs efficiently at the SiNC and polymer interfaces, however, electron transportation *via* particle-to-particle, or hoping mechanism, may be disturbed unless the electron transport percolation pathway is created. Presumably charge recombination at the interface occurs rather dominantly which increases the internal resistance of the photoactive layer, yielding a small value of FF. A detailed analysis of the internal morphology of the SiNC network and the optimization of the complemental polymer matrix is the critical issue for the elucidation of the charge transport mechanism and thus the further improvement of PV performance.

**Table 1:** Performance parameters of hybrid photovoltaics corresponding to *J*–*V* characteristics (1 sun)

Device configuration	*J*_SC_ [mA cm^−2^]	*V*_OC_ [V]	FF [%]	PCE [%]
As-H:SiNCs/PTB7	9.39	0.67	38.99	2.44
H:SiNCs (200 °C)/PTB7	8.24	0.67	35.37	1.96
H:SiNCs (400 °C)/PTB7	0.24	0.51	28.97	0.03
TiO_2_-NCs/PTB7	0.57	0.19	33.94	0.04
As-H:SiNCs/PTB7-Th	9.70	0.66	46.71	3.01

**Fig. 6:**
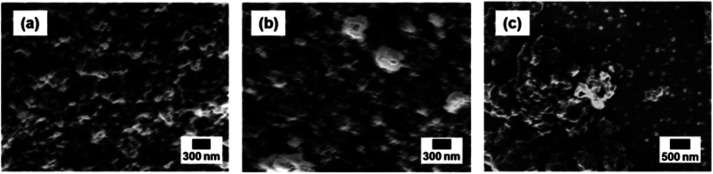
Top view SEM micrographs of SiNCs/PTB7 photoactive layer: (a) as-etched SiNCs; (b) low-defect SiNCs (200 °C annealing); (c) high-defect SiNCs (400 °C annealing).

The morphology of the photoactive layers was investigated using scanning electron microscopy as shown in Fig. 6 (SEM, JSM-7800; JEOL Ltd.). The morphology plays a key role in the photoactive layer because photogenerated excitons have a high binding energy, and the diffusion length in the polymer matrix is generally as short as 10 nm. As-etched SiNCs were moderately blended with PTB7 without remarkable aggregation. This morphology provided better exciton dissociation interfaces and electron transport pathways, resulting in a higher *J*_SC_, but low fill factor (Fig. 6(a)). In the case of low-defect SiNCs, some aggregation of SiNCs was observed all over the layer (Fig. 6(b)). Although low-temperature (200 °C) annealing decreases SiNC defects effectively, the agglomeration of SiNCs is likely to occur which deteriorates PV performance. In the case of high-defect SiNCs, we observed the remarkable aggregation of SiNCs by naked eyes, which was further confirmed by the SEM micrograph as shown in Fig. 6(c). The size of the aggregations (*ca.* 1 μm) was larger than the thickness of the photoactive layer, which is clearly detrimental for the generation and transportation of photogenerated carriers.

**Fig. 7:**
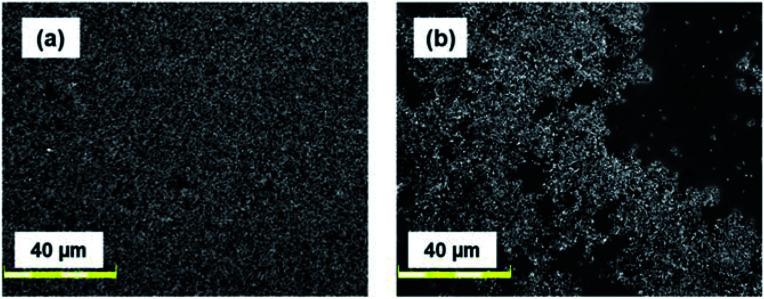
Laser scanning microscopy images of HPVs based on (a) as-etched SiNCs; (b) TiO_2_ nanocrystals.

As a comparison, titanium oxide nanocrystals (TiO_2_, 5 nm, anatase; EM Japan) were examined, and their photovoltaic performance was compared with that of SiNC-HPVs. TiO_2_ has been widely used as an n-type semiconducting material with efficient electron transport mobility in numerous electronic devices. The energy diagram is compared with that of the SiNCs in Fig. 2. The energy alignment of TiO_2_ is better than that of SiNCs because the energy offset between the TiO_2_ conduction band edge and the LUMO level of PTB7 is larger than that of the SiNCs, enabling efficient exciton dissociation at their interfaces. Moreover, the TiO_2_ valence band edge is located suitably so that hole transport to the aluminium electrode is prevented. However, the TiO_2_-HPVs showed an extremely low photovoltaic performance as shown in Fig. 5 and Table 1. Fig. 7 shows that the dispersion state of TiO_2_ nanocrystals over the polymer matrix is insufficient, and a bulk-heterojunction-type structure is not fabricated. Modification of TiO_2_ surface with organic ligands would improve the dispersion of TiO_2_ nanoparticles in the solvent for forming a better photoactive layer.^51–53^ However, in electronic device applications, organic ligands are not suitable because the surface ligand hinders carrier mobility between particles, decreasing the device performance.^1^

**Fig. 8:**
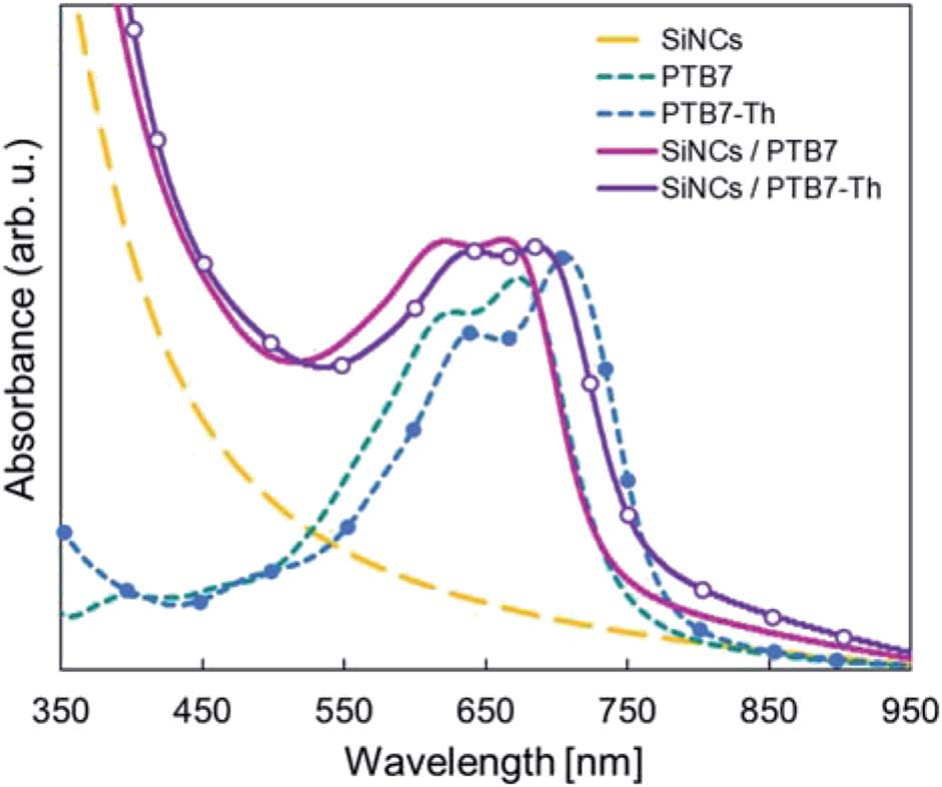
Absorbance spectra of SiNCs, PTB7, PTB7-Th, and their blended films.

As another choice for an electron donor material, PTB7-Th should be more suitable with an effective band gap of 1.57 eV. Optical absorption spectra were measured for SiNCs, PTB7, PTB7-Th, and their blended films as the photoactive layer after spin-coating on a glass substrate using a calibrated UV spectrophotometer (UV-1800; Shimadzu) (Fig. 8). The SiNCs/PTB7-Th blended film had a larger absorption spectrum than the SiNCs/PTB7 blended film in the longer wavelength region, which is expected to produce a higher *J*_SC_ value. The as-etched SiNCs/PTB7-Th device was fabricated, and the *J*–*V* characteristics and performance parameters are summarized in Fig. 7 and Table 1. The PCE reached up to 3.0%: the *J*_sc_ increased slightly, but the fill factor improved remarkably.

### 3.3 Device performance under standard indoor light

**Fig. 9:**
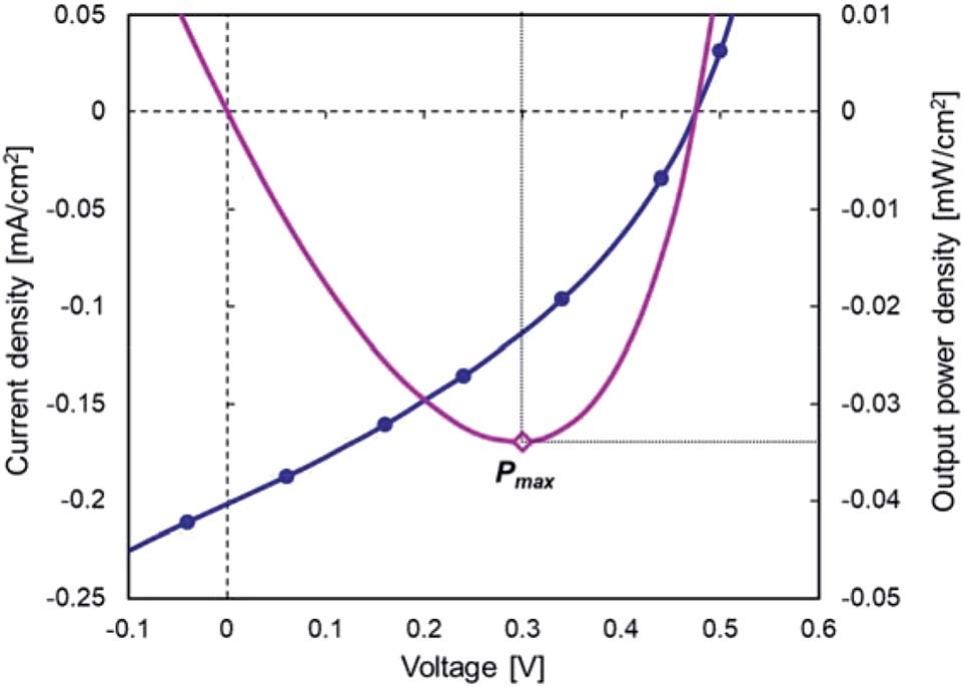
*J*–*V* characteristics of SiNC-HPVs under simulated standard indoor light (1000 lx).

**Fig. 10:**
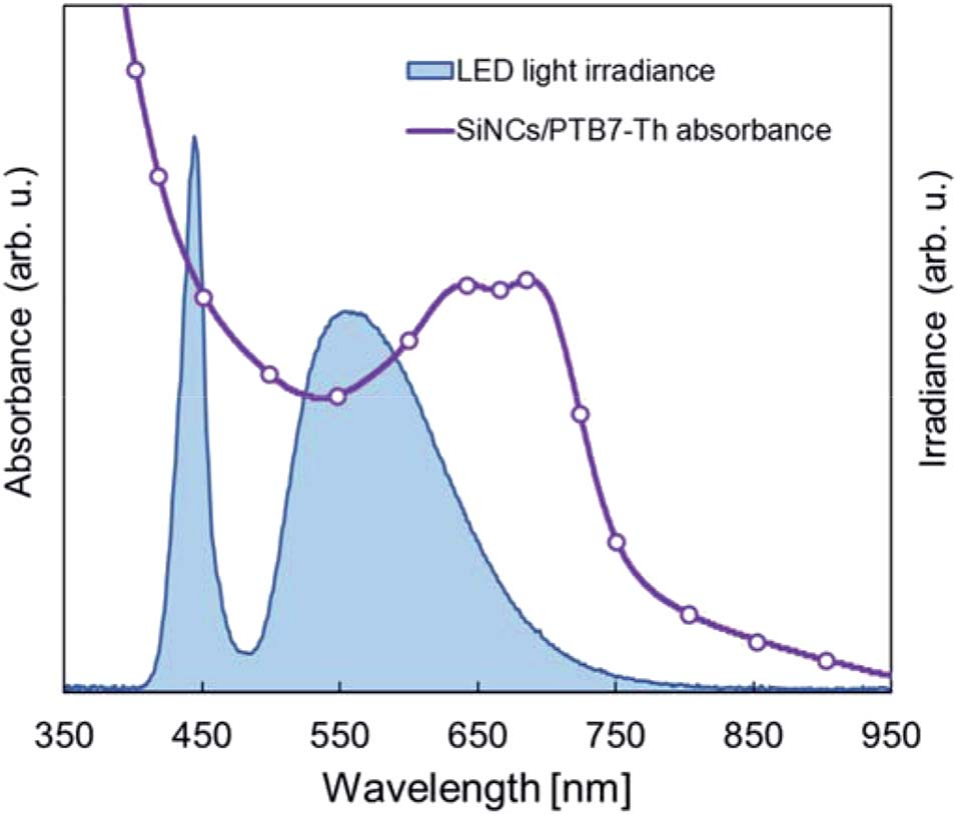
Irradiance spectrum of the LED light source *vs.* SiNCs/PTB7-Th absorbance spectrum.

Device performance measurement under standard indoor light at 1000 lx was performed as a reference to the illuminance in a typical office setting. The as-etched SiNCs/PTB7-Th device was used for performance measurements. *J*–*V* characteristics are shown in Fig. 9, and the corresponding performance parameters are listed in Table 2. The maximum output power density (*P*_max_) was 34.0 μW cm^−2^ with a *J*_SC_ of 0.195 mA cm^−2^, *V*_OC_ of 0.47 V and FF of 35.48%, while the PCE was 9.71%. The irradiance of the LED light source at 1000 lx was 0.35 mW cm^−2^, and the PCE was estimated by dividing the *P*_max_ of the device by the irradiance of the LED light source. The device shows a low *J*_SC_ since the illuminance of the LED light source is *ca.* 1/120 of that of the solar simulator (1 sun). The irradiance spectra of the LED light source are shown in Fig. 10. The photoactive layer has a complimentary light absorption spectrum shorter than 800 nm, which would sufficiently convert the entire range of indoor light spectra into photocurrent. The SiNC absorbance spectrum matches the irradiance sharp peak at 440 nm, and the PTB7-Th absorbance spectrum covers the entire irradiance spectrum range between 500 nm and 700 nm. Consequently, a PCE of 9.71% under the standard indoor light was obtained since the energy loss between the photoactive layer light absorption spectrum and light source wavelength was reduced. The power consumption of a low-powered IoT device typically ranges between 20 μW and 10 mW,^31,54^ and SiNC-HPVs enables sufficient power supply to the IoT device by designing an optimum system structure. A typical fullerene-based OPV has been reported to exceed a PCE of 10% under a simulated indoor light source.^33–39^ Also, the SiNC-HPVs can be a potential energy harvesting device for indoor IoT applications.

**Table 2:** Performance parameters of hybrid photovoltaics corresponding to *J*–*V* characteristics (indoor light; 1000 lx)

Device configuration	*J*_SC_ [mA cm^−2^]	*V*_OC_ [V]	FF [%]	*P*_max_ [μW cm^−2^]	PCE [%]
As-H:SiNCs/PTB7-Th	0.195	0.47	35.48	34.0	9.71

## 4. Conclusion

This paper demonstrated SiNC-HPVs and obtained a PCE up to 3.0% under standard solar irradiation (1 sun). Additionally, the use of SiNC-HPVs for indoor applications was examined, and PCE under the simulated standard indoor light reached 9.7% because of the suitability of the light absorption spectrum and light source wavelength due to SiNCs. This value is more than three times as high as that measured under standard solar irradiation, indicating the potential use of SiNC-HPVs for indoor energy harvesting systems of IoT applications. Moreover, amount of SiNC defects was reduced by thermal annealing at temperatures below 200 °C while suppressing hydrogen desorption on the SiNC surface. However, thermally treated SiNCs tend to form aggregates in the polymer matrix, and the device performance slightly deteriorates. Further performance improvement would be expected when the agglomeration of SiNCs is suppressed during low-temperature annealing and forms better nanostructured SiNCs/polymer blended photoactive layer with improved FF values.

## Conflicts of interest

There are no conflicts to declare.

## Supplementary Material

RA-010-D0RA00804D-s001
