# Supplementary material for: Silicon nanocrystal hybrid photovoltaic devices for indoor light energy harvesting
Source: RSC Adv. 2020 Mar 27;10(21):12611–8. doi: 10.1039/d0ra00804d (PMC9051256; doi:10.1039/d0ra00804d)
Supplement: RA-010-D0RA00804D-s001 [file RA-010-D0RA00804D-s001.pdf]

Electronic Supplementary Material (ESI) for RSC Advances.
This journal is © The Royal Society of Chemistry 2020

Electronic supplementary information of

Silicon nanocrystal hybrid photovoltaic devices for indoor light energy harvesting

Munechika Otsuka [a], Yuki Kurokawa [b], Yi Ding [c], Firman Bagja Juangsa [d], Shogo Shibata [a], Takehito Kato [b], Tomohiro Nozaki [a, *]

[a] School of Engineering, Tokyo Institute of Technology, 2-12-1 O-okayama, Meguro-ku, Tokyo, 152-8550, Japan

[b] Department of Mechanical Engineering, National Institute of Technology, Oyama College, 771 Nakakuki, Oyama, Tochigi, 323-0806, Japan

[c] Institute of Photoelectronic Thin Film Devices and Technology, Nankai University, Tianjin 300071, PR China

[d] Faculty of Mechanical and Aerospace Engineering, Institut Teknologi Bandung, Ganesha street no. 10, Bandung, 40132, Indonesia

* E-mail: nozaki.t.ab@m.titech.ac.jp

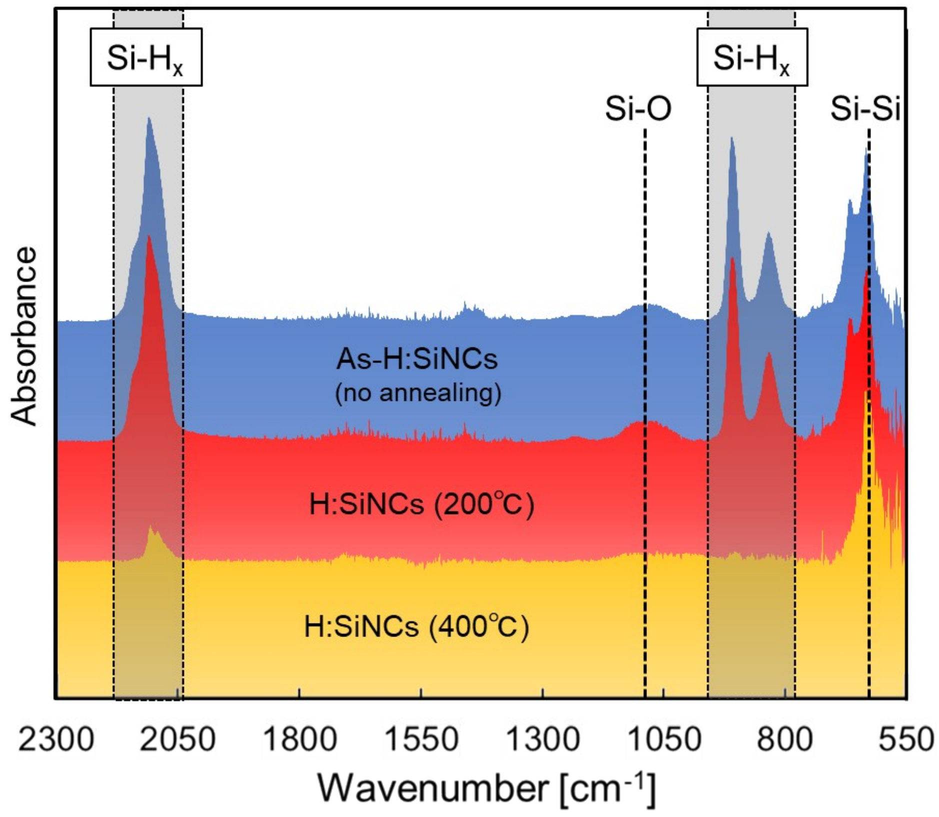

Figure S1. FTIR spectra of SiNCs before and after thermal annealing; as-H:SiNCs (no annealing), H:SiNCs (200 °C for 1 hour) and H:SiNCs (200 °C for 1 hour)

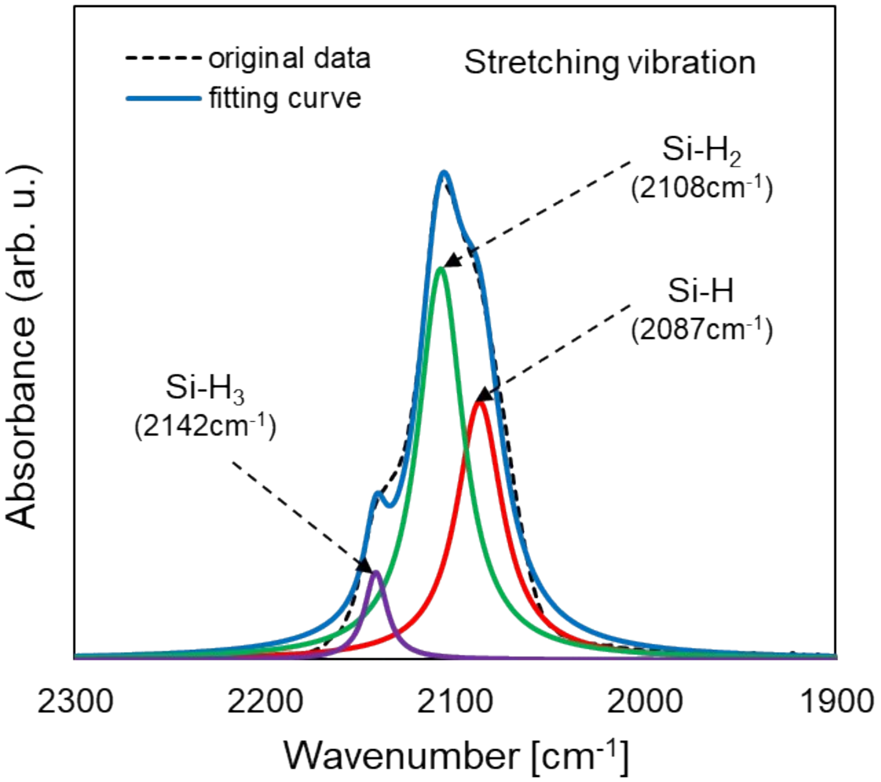

Figure S2. Deconvolution of SiH_n bonds on the as-H:SiNC (no annealing) surface
